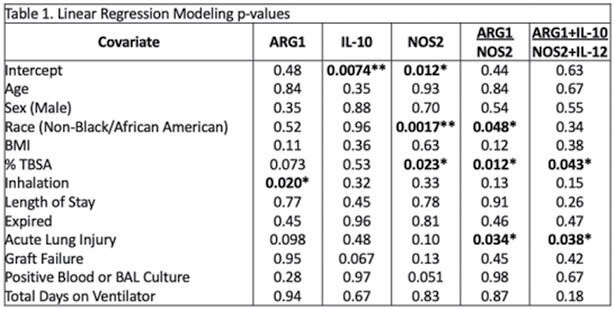# 41 Peripheral Mononuclear Cell Expression of IL-10, IL-12, ARG1, & NOS2 Correlates with Outcomes After Burn

**DOI:** 10.1093/jbcr/irac012.044

**Published:** 2022-03-23

**Authors:** Cressida Mahung, Wesley H Stepp, Madison Malfitano, Shannon Wallet, Laura Y Zhou, Haibo Zhou, Robert Maile

**Affiliations:** University of North Carolina at Chapel Hill, Washington, District of Columbia; UNC, CARRBORO, North Carolina; UNC Health, Chapel hill, North Carolina; University of North Carolina at Chapel Hill, Chapel Hill, North Carolina; University of North Carolina, Chapel Hill, North Carolina; University of North Carolina at Chapel Hill, Chapel Hill, North Carolina; UNC, Chapel Hill, North Carolina

## Abstract

**Introduction:**

In the burned patient, clinical outcomes are inextricably linked with immune function. Patients are subject to an early pro-inflammatory response and a subsequent compensatory anti-inflammatory response syndrome. This dysregulation can lead to infection, multiple organ dysfunction syndrome, and death. Despite continuing efforts, a profile of immune gene expression from burn patients that can be transformed into an “immune suppression index”, which accurately reflects the underlying degree of immune insult and significantly correlates with the degree compromise, has yet to be developed. The development of such an approach that can predict graft failure, susceptibility to infection, length-of-stay in the hospital, and/or that can inform a provider on how to better manage the timing of surgical interventions would be transformative.

**Objective:**

To determine a set of peripheral biomarkers that correlates with clinical outcomes of burn patients.

**Methods:**

This observational study enrolled two participant cohorts within a single burn center. Initial unbiased analysis compared 23 burn patients and 6 healthy controls. Confirmatory outcomes analysis was performed in 109 burn patients and 19 healthy controls. We employed multiplex gene expression analysis to identify differential peripheral blood mononuclear cells (PBMC) immune gene expression. qPCR was used to validate these findings, identify, and model associations with outcomes.

**Results:**

We identified 149 genes with a significant difference in expression within PBMCs from burn patients compared to controls (Figure 1a). Pathway analysis identified pathways related to IL-10 and inducible nitric oxide synthase (iNOS) signaling (Figure 1b). qPCR analysis of IL-10, IL-12, arginase-1 (ARG1), and iNOS demonstrated that burn injury was associated with increased expression of ARG1 and IL-10, and decreased expression of NOS2 and IL-12. Burn severity, acute lung injury (ALI), development of infection, failure of skin autograft, and mortality significantly correlated with expression of one or more of these genes. Ratios of IL-10/IL-12, ARG1/NOS2 and (ARG1+IL-10)/(NOS2+IL-12) transcript levels further improved the correlation with outcomes. A multivariate regression model, adjusting for confounders, demonstrated that (ARG1+IL-10)/(NOS2+IL-12) significantly correlated with burn severity and development of ALI (Table 1).

**Conclusions:**

We present a robust model to predict patient outcomes early after burn injury using non-invasive methods, allowing early identification of underlying immune dysfunction.